# Associations of air pollution exposure with blood pressure and heart rate variability are modified by oxidative stress genes: A repeated-measures panel among elderly urban residents

**DOI:** 10.1186/s12940-016-0130-3

**Published:** 2016-03-25

**Authors:** Kyoung-Nam Kim, Jin Hee Kim, Kweon Jung, Yun-Chul Hong

**Affiliations:** Department of Preventive Medicine, Seoul National University College of Medicine, Seoul, Republic of Korea; Department of Bioscience and Bioengineering, Sejong University, Seoul, Republic of Korea; Seoul Metropolitan Institute of Public Health and Environment, Seoul, Republic of Korea; Institute of Environmental Medicine, Medical Research Center, Seoul, Republic of Korea; Environmental Health Center, Seoul National University College of Medicine, Seoul, Republic of Korea

**Keywords:** Air pollution, Heart rate variability, Blood pressure, Oxidative stress, Genetic factors

## Abstract

**Background:**

Oxidative stress has been suggested as a major cause of elevated blood pressure (BP) and reduced heart rate variability (HRV) due to air pollution. We hypothesized that the associations of air pollution exposure with BP and HRV are modified by oxidative stress gene polymorphisms.

**Methods:**

Between 2008 and 2010, we conducted up to 5 surveys of 547 elderly participants, measured their BP and HRV, and genotyped 47 single nucleotide polymorphisms (SNPs) in 18 oxidative stress genes. Linear mixed models were constructed to evaluate the associations of particulate matter ≤10 μm, nitrogen dioxide, and sulfur dioxide with BP and HRV, as well as the modifications of these associations by the genotyped SNPs.

**Results:**

Single-SNP analyses revealed interactions between air pollution and 15 SNPs (for BP) and 33 SNPs (for HRV) (all, *P* for interaction < 0.05). When we generated genetic risk scores for BP and HRV, using the SNPs with interactions in the single-SNP models, we found that associations of air pollution exposure with BP and HRV were modified by the genetic risk scores (*P* for interaction < 0.05).

**Conclusions:**

These results strongly suggest that the associations of air pollution with BP and HRV are mediated by oxidative stress pathways.

**Electronic supplementary material:**

The online version of this article (doi:10.1186/s12940-016-0130-3) contains supplementary material, which is available to authorized users.

## Background

Air pollution exposure is associated with increased cardiovascular morbidity and mortality [1], which are one of the most prominent health outcomes related to air pollution [2]. Previous studies have suggested that changes in blood pressure [3] and heart rate variability [4] could be induced by air pollution, and these changes may be responsible for the reported increase in cardiovascular morbidity and mortality due to air pollution [5]. Oxidative stress has been indicated as a major cause of the associations of air pollution exposure with increased blood pressure and decreased heart rate variability [6, 7]. Therefore, it is biologically plausible that the associations of air pollution with blood pressure and heart rate variability are modified by a genetic predisposition to oxidative stress [8].

Despite the importance of gene-environment interactions for identifying and assessing potential mechanistic pathways for health conditions [9, 10], few studies have evaluated the air pollution-gene interaction with regard to blood pressure or heart rate variability, and their results have been inconsistent [5, 11–18]. Furthermore, these studies have been conducted mostly in a few populations of middle-aged or elderly Caucasians, which limits the external generalizability and does not exclude the possibility of random error or population stratification. In addition, previous studies have focused on individual single nucleotide polymorphisms (SNPs) or genes, lowering the ability to comprehensively assess the oxidative stress pathways with regard to blood pressure and heart rate variability, because single SNPs typically have small effects and may affect specific phenotypes cumulatively by working together with other SNPs.

Therefore, in the present study, we hypothesized that oxidative stress-related genetic polymorphisms could modify the associations of air pollution exposure with blood pressure and heart rate variability. We evaluated the hypothesis using a repeated-measures panel of elderly urban residents in Seoul, Korea, and used genetic risk scores instead of individual SNPs or genes to summarize the cumulative effects of genetic polymorphisms in the oxidative stress pathway as a potential mechanisms behind the associations of air pollution exposure with blood pressure and heart rate variability [19].

## Methods

### Study design and population

The Korean Elderly Environmental Panel (KEEP) study was a community-based repeated-measures study that aims to evaluate the relationship between environmental risk factors and adverse health outcomes in an elderly population. We recruited 560 non-institutionalized elderly individuals who regularly visited a community welfare center that is located in Seongbuk-Gu district, Seoul, Republic of Korea. The inclusion criteria were an age of ≥60 years and the ability to communicate and follow the instructions of the survey staff. Information regarding the participants’ sociodemographic characteristics, lifestyle, and medical history was obtained using a structured questionnaire, which was administered by trained interviewers at the baseline survey. Between August 2008 and August 2010, we conducted up to 5 surveys for each participant with measurement of blood pressure, heart rate variability, and anthropometric parameters.

Among the 560 participants, we subsequently excluded 7 individuals for having no blood pressure measurement and 6 individuals for having no heart rate variability information. Therefore, we included data from 547 participants in the final analyses. All participants provided their written informed consent, and the study protocol was approved by the ethical review board at Seoul National University Hospital (C-704-040-205).

### Air pollutant concentrations and meteorological factors

We estimated the individuals’ exposures to air pollutants, such as particulate matter ≤10 μm (PM_10_), nitrogen dioxide (NO_2_), and sulfur dioxide (SO_2_), using 24-h monitoring data that were obtained from the Korea National Institute of Environmental Research, Incheon, Republic of Korea. We used the daily mean values for PM_10_, NO_2_, and SO_2_ from the monitoring center that was nearest to each participant’s residence. The mean distance between the monitoring centers and the participants’ residences was <1 km. Detailed information regarding the measurement methods has been described elsewhere [20]. Daily mean temperatures and dew point temperatures were measured at the monitoring center that was nearest to each participant’s residence, and these data were obtained from the Korea Meteorological Administration, Seoul, Republic of Korea. Next, we calculated the apparent temperature using the following formula [21–24]: Apparent temperature = –2.653 + [0.994 × daily mean temperature (°C)] + (0.0153 × [daily dew point temperature (°C)]^2^).

### Genetic polymorphisms

We used the QIAamp DNA Blood Mini Kit (Qiagen, Valencia, CA, USA) to extract genomic DNA from samples of the participants’ peripheral blood lymphocyte. We analyzed 18 genes (AhR, ANKK1, CAT, COMT, CYP1A1, CYP1B1, CYP2B6, EPHX1, GSTP1, HSPA1L, MPO, MTHFR, NAT2, NOS3, NQO1, PON1, PTGS2, and SOD2) that are related to oxidative stress, and genotyped 47 SNPs from these genes. The SNPs were selected based on a priori knowledge of being associated with oxidative stress status, a previous study from the National Center for Biotechnology Information, as well as minor allele frequencies of ≥5 % for the Japanese and Chines populations in the HapMap (http://hapmap.ncbi.nlm.nih.gov/) to consider public health implications. Polymorphisms in ANKK1, CAT, CYP1B1, EPHX1, HSPA1L, MPO, NOS3, PON1, PTGS2, and SOD2 were identified using a TaqMan fluorogenic 5’ nuclease assay (ABI, Foster City, CA, USA); polymorphisms in AhR, COMT, CYP1A1, CYP2B6, MTHFR, NAT2, and NQO1 were identified using the Sequenom Mass ARRAY platform [25]; and polymorphisms in GSTP1 were identified using a multiplex polymerase chain reaction method [26]. Among the 47 genotyped SNPs, we excluded one SNP (rs2965753) from the present analyses because it was not within the Hardy-Weinberg equilibrium (*P* = 9.3126 × 10^-7^ using the chi square test).

### Blood pressure and heart rate variability

Blood pressure and heart rate variability were measured between 10:00 AM and 12:00 PM. After ≥10 min of rest, a trained medical technologist measured the participant’s blood pressure using an autonomic sphygmomanometer (HEM-780; Omron, Kyoto, Japan). Once the first measurement was finished, the participant was asked to sit and rest for another 10 min, and their blood pressure was subsequently re-measured. We averaged the two blood pressure measurements and used the mean value as a main outcome variable. We also calculated the mean arterial pressure by adding one-third of the systolic blood pressure and two-thirds of the diastolic blood pressure.

To measure heart rate variability, participants were asked to attach 3 limb leads to both wrists and their left ankle, and to relax in the seated position for ≥5 min. Heart rate variability was automatically analyzed via electrocardiography using a heart rate variability analyzing device (SA-3000P; Medicore, Seoul, Republic of Korea). We analyzed the standard deviation of normal-to-normal intervals (SDNN) and the root mean square of successive differences (RMSSD) for time domain measures, and low frequency power (0.04–0.15 Hz, LF) and high frequency power (0.15–0.40 Hz, HF) for frequency domain measures.

### Statistical analysis

We log-transformed the variables that exhibited log-normal distributions (e.g., SDNN, RMSSD, LF, and HF) and used these values for our analyses. In the KEEP study, exposures and outcomes were repeatedly measured for each participant up to 5 times, and we constructed the long format data set, which stacks information obtained from each survey in the row. To consider intra-individual correlation due to repeated-measures data structure, linear mixed models were constructed. We evaluated the associations of exposure to air pollutants (PM_10_, NO_2_, and SO_2_) with blood pressure (systolic blood pressure, diastolic blood pressure, and mean arterial pressure) and heart rate variability (SDNN, RMSSD, LF, and HF) using these models. We also applied daily lag structures up to 3 days, and reported the results from the models with the best fit (which were determined using the Akaike information criterion). After we analyzed the minor allele frequency and Hardy-Weinberg equilibrium for each SNP, we added interaction terms for each air pollutant and the SNP to the main models, which contained lower order terms and covariates to assess the interaction. All SNPs were modeled as an additive model, which is known to provide good performance, even in cases where the true genetic model is not known [27, 28]. The general form of the linear mixed models used in this analysis is presented in the Additional file 1.

We calculated the genetic risk scores for blood pressure and for heart rate variability by summing the number of risk alleles for the SNPs that exhibited interactions (*P* value for interaction < 0.05) with any air pollutant with regard to blood pressure or heart rate variability. To satisfy the assumption that each SNP in the genetic risk score was independently associated with risk, we estimated the linkage disequilibrium between the SNPs within the same gene by calculating |D’| values and selected one SNP within each linkage disequilibrium block. Furthermore, we categorized the genetic risk scores for blood pressure and heart rate variability into tertiles. We then assessed the association between air pollution exposure and the outcome of interest within each tertile using linear mixed models, after we had performed nonparametric analyses using the generalized additive mixed models (see Additional file 1). Heterogeneity in the associations according to the genetic risk scores was assessed by adding and testing the product term of the tertile score and each air pollutant.

All models were adjusted for covariates that were selected a priori: age (years), sex, smoking status (current smoker, ex-smoker, non-smoker, did not answer), alcohol drinking (current drinker, non-drinker, did not answer), body mass index (kg/m^2^), hypertension medication (no, yes), and apparent temperature. All covariates except for body mass index and apparent temperature were included in the models as time-independent. Although demographic and lifestyle factors may not influence air pollution exposure, we included them to block any potential backdoor path [29]. All analyses were performed using SAS software (version 9.4; SAS Institute Inc., Cary, NC, USA), R software (version 3.1.0; Comprehensive R Archive Network: http://cran.r-project.org), and Haploview software (version 4.2; http://www.broadinstitute.org/haploview). All *P*-values were two-sided.

We performed several sensitivity analyses. First, we evaluated the interaction of each air pollutant with each SNP using the 2-df joint test, which simultaneously tests the main effect of the SNP and its interaction with the environmental factor [30]. To perform the 2-df joint test, we performed a likelihood ratio test using a full model that included terms for the SNP, air pollutant, their interaction, and any covariates, as well as a nested model that excluded the SNP and interaction terms, using the SAS macro MIXED_FIT and R package lme4. Second, we determined the genetic risk scores using different set of SNPs, which were selected from each linkage disequilibrium block, and assessed whether the results were robust. Third, we weighted the follow-up observations using the inverse probability of having a follow-up response, in order to reduce the potential selection bias that is caused by non-random loss to follow-up [31]. Logistic regression was performed to estimate the probability of a follow-up, and the covariates included age, sex, body mass index, years of schooling, blood pressure, season, and outdoor temperature at the prior visit. We gave a weight of 1 to the first observation and the inverse probability of follow-up to each follow-up observation [32].

## Results

Table 1 shows the participants’ baseline characteristics. All participants were ≥60 years old, and the mean age was 70.7 years. Among the 547 individuals, 404 (73.9 %) were women, 467 (85.4 %) were non-smokers, and 412 (75.3 %) were non-drinkers. The average body mass index was 24.7 kg/m^2^.

**Table 1:** Characteristics of the Participants at Enrollment in the Korean Elderly Environmental Panel Study (2008–2010)

	All (*n* = 547)	Men (*n* = 143)	Women (*n* = 404)
Age (year)	70.7 (5.2)	71.4 (4.4)	70.4 (5.5)
Height (cm)	154.7 (7.7)	164.3 (5.3)	151.3 (5.0)
Weight (kg)	59.3 (9.0)	65.7 (9.8)	57.0 (7.4)
Smoking status			
Current smoker	30 (5.5)	29 (20.3)	1 (0.3)
Ex-smoker	36 (6.6)	32 (22.4)	4 (1.0)
Non-smoker	467 (85.4)	79 (55.2)	388 (96.0)
Did not answer	14 (2.6)	3 (2.1)	11 (2.7)
Alcohol consumption			
Current drinker	118 (21.6)	77 (53.9)	41 (10.2)
Nondrinker	412 (75.3)	61 (42.7)	351 (86.9)
Did not answer	17 (3.1)	5 (3.5)	12 (3.0)
Blood pressure (mmHg)			
Systolic blood pressure	131.8 (16.7)	130.8 (17.0)	132.1 (16.6)
Diastolic blood pressure	74.5 (9.9)	73.9 (10.2)	74.7 (9.8)
Mean arterial pressure	93.6 (11.5)	92.9 (11.9)	93.8 (11.4)
Heart rate variability			
SDNN (ms)	26.9 (1.7)	25.4 (1.7)	27.4 (1.6)
RMSSD (ms)	20.5 (2.0)	18.4 (2.1)	21.3 (1.9)
LF (ms^2^)	90.83 (3.5)	79.0 (3.5)	95.4 (3.5)
HF (ms^2^)	66.9 (3.8)	50.6 (4.1)	73.8 (3.6)

*HF* high frequency power for frequency domain, *LF* low frequency power for frequency domain, *RMSSD* root mean square of successive differences for time domain, *SDNN* standard deviations of normal-to-normal intervals for time domain

Categorical data are shown as *n* (%) and continuous data as mean (SD), except for heart rate variability, which is presented as geometric mean (geometric SD)

Table 2 presents the air pollutant levels and meteorological factors on the days when the health examinations were conducted. The mean concentrations of PM_10_, NO_2_, and SO_2_ were 42.6 μg/m^3^, 36.5 ppb, and 4.0 ppb, respectively. The mean temperature and dew point were 16.9 °C and 6.2 °C, respectively. The air pollutant levels and meteorological factors on the health examination days were similar over the 3 previous lag days (data not shown).

**Table 2:** Air Pollutant Levels and Meteorological Factors in Seongbuk-Gu (Seoul, Republic of Korea) on the Survey Days, Korean Elderly Environmental Panel Study, 2008–2010

	Mean (SD)	Median	Range	IQR
PM_10_ (μg/m^3^)	42.6 (24.7)	37.1	7.6, 151.3	25.4
NO_2_ (ppb)	36.5 (12.6)	34.9	9.8, 81.0	16.8
SO_2_ (ppb)	4.0 (2.1)	3.5	1.0, 13.9	2.4
Mean temperature (°C)	16.9 (9.0)	18.5	-7.2, 29.2	15.0
Dew point (°C)	6.2 (10.8)	7.7	-25.6, 21.9	17.2

*IQR* interquartile range, *NO*_2_ nitrogen dioxide, *PM*_10_ particulate matter ≤10 μm, *SD* standard deviation, *SO*_2_ sulfur dioxide

An interquartile-range increase in the air pollutants (PM_10_, NO_2_, and SO_2_) was positively associated with systolic blood pressure (PM_10:_ β = 0.93, 95 % confidence interval [CI]: 0.23, 1.63; NO_2_: β = 0.96, 95 % CI: 0.18, 1.74; SO_2_: β = 1.60, 95 % CI: 0.79, 2.42), diastolic blood pressure (PM_10_: β = 0.62, 95 % CI: 0.20, 1.03; NO_2_: β = 0.77, 95 % CI: 0.31, 1.24; SO_2_: β = 0.74, 95 % CI: 0.26, 1.23), and mean arterial pressure (PM_10_: β = 0.71, 95 % CI: 0.22, 1.19; NO_2_: β = 0.82, 95 % CI: 0.28, 1.36; SO_2_: β = 1.02, 95 % CI: 0.46, 1.58). However, air pollution exposure was not associated with heart rate variability (Table 3). In multiple pollutant models which include all air pollutants and covariates, SO_2_ levels were still associated with systolic blood pressure (β = 1.43, 95 % CI: 0.60, 2.26), diastolic blood pressure (β = 0.59, 95 % CI: 0.09, 1.09), and mean arterial pressure (β = 0.86, 95 % CI: 0.29, 1.44), while other associations were not found (data not shown).

**Table 3:** Associations of PM_10_, NO_2_, and SO_2_ with Blood Pressure and Heart Rate Variability per Interquartile-Range Increase in Air Pollutant Concentration Using Linear Mixed Models, Korean Elderly Environmental Panel Study, 2008–2010

	PM_10_			NO_2_			SO_2_		
	No. of Obs.	Estimate	95 % CI	No. of Obs.	Estimate	95 % CI	No. of Obs.	Estimate	95 % CI
SBP	1719	0.93	0.23, 1.63	1719	0.96	0.18, 1.74	1719	1.60	0.79, 2.42
DBP	1719	0.62	0.20, 1.03	1719	0.77	0.31, 1.24	1719	0.74	0.26, 1.23
MAP	1719	0.71	0.22, 1.19	1719	0.82	0.28, 1.36	1719	1.02	0.46, 1.58
SDNN	1719	-0.01	-0.03, 0.02	1719	-0.02	-0.05, 0.01	1719	0.01	-0.02, 0.03
RMSSD	1718	-0.01	-0.04, 0.02	1718	-0.02	-0.05, 0.02	1718	0.01	-0.02, 0.05
LF	1719	0.004	-0.06, 0.06	1719	-0.02	-0.10, 0.05	1719	0.04	-0.02, 0.11
HF	1719	-0.03	-0.09, 0.03	1719	-0.05	-0.13, 0.02	1719	-0.004	-0.07, 0.06

*CI* confidence interval, *DBP* diastolic blood pressure, *HF* high frequency power for frequency domain, *LF* low frequency power for frequency domain, *MAP* mean arterial pressure, *No. of Obs.* number of observations used in the analysis, *NO*_2_ nitrogen dioxide, *PM*_10_ particulate matter ≤10 μm, *RMSSD* root mean square of successive differences for time domain, *SBP* systolic blood pressure, *SDNN* standard deviations of normal-to-normal intervals for time domain, *SO*_2_ sulfur dioxide

We constructed separate models to assess the interaction of 46 SNPs in 18 genes with air pollution exposure. Among these models, 15 SNPs exhibited interactions (*P* value for interaction < 0.05) with the air pollutants with regard to blood pressure. We selected 11 SNPs (rs4646421, rs1801133, rs2917670, rs2066853, rs4680, rs854560, rs5277, rs769218, rs1799983, rs7830, and rs2234922) from these 15 SNPs, because we observed linkage disequilibrium between rs4646422 and rs4646421 (|D’| = 0.98), rs1437135 and rs2917670 (|D’| = 0.93), rs769218 and rs769217 (|D’| = 1), and rs1799983 and rs2853796 (|D’| = 0.94). The genetic risk score for blood pressure was calculated by summing the number of risk alleles for the 11 SNPs. The genetic risk score for heart rate variability was calculated using 17 SNPs (rs1695, rs4646422, rs2855658, rs10012, rs1801133, rs1800566, rs3745274, rs1799931, rs854560, rs662, rs2758331, rs5277, rs769217, rs2227956, rs2853796, rs7830, and rs1051740) that were selected using the same procedure. In the present study, we did not use weighted genetic risk scores due to methodological difficulties in considering and summarizing different interaction effects among SNPs in gene-environment interaction study.

**Fig. 1:**
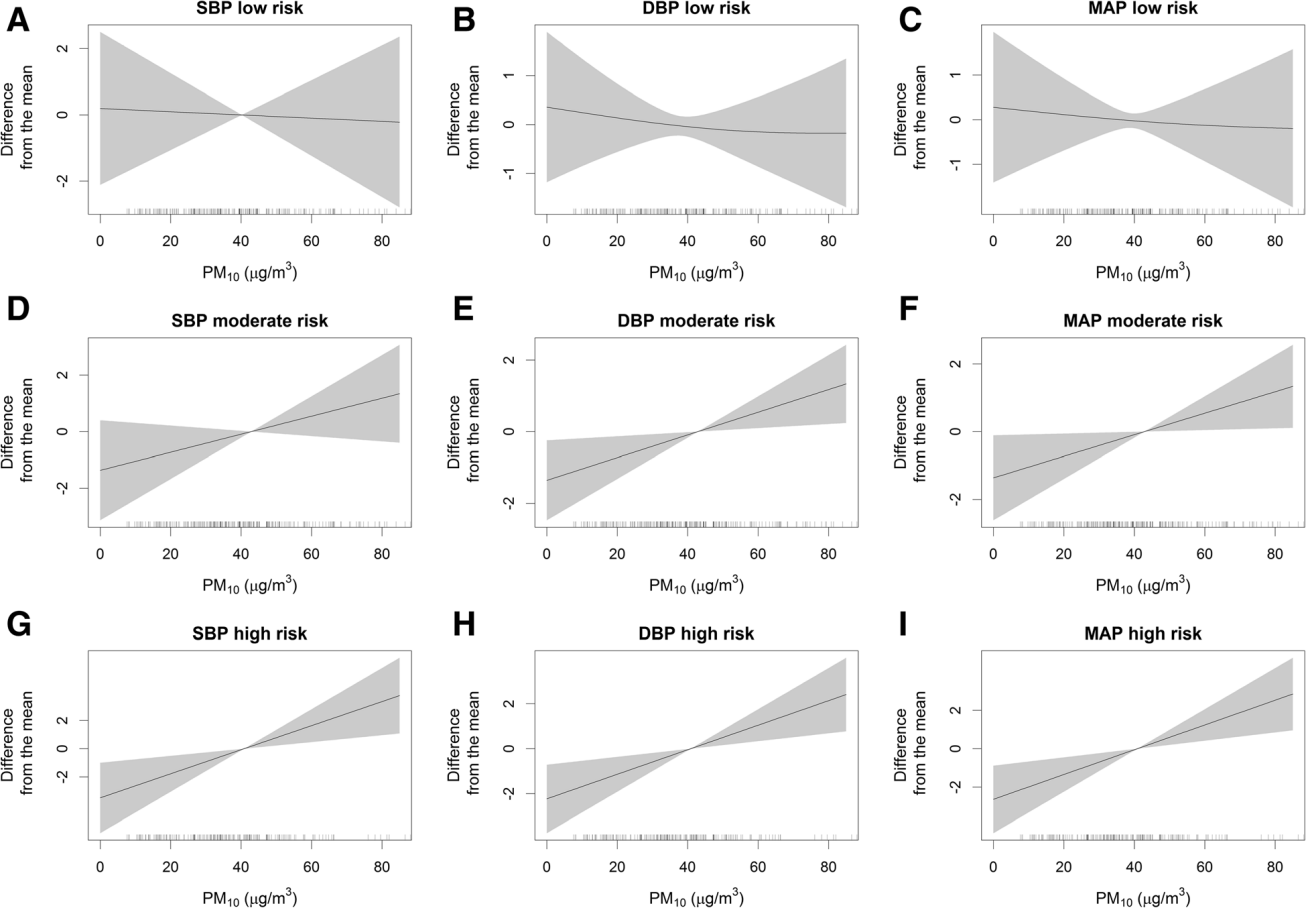
Associations between ambient PM_10_ concentration and blood pressure in generalized additive mixed models, stratified according to tertiles of genetic risk score for blood pressure, Korean Elderly Environmental Panel Study, 2008–2010. Models were adjusted for age, sex, smoking status, alcohol drinking, body mass index, hypertension medication, and apparent temperature. The plots show the associations of ambient PM_10_ concentration with systolic blood pressure (**a**), diastolic blood pressure (**b**), and mean arterial blood pressure (**c**) in the low genetic risk score group; the associations with systolic blood pressure (**d**), diastolic blood pressure (**e**), and mean arterial blood pressure (**f**) in the moderate genetic risk score group; and the associations with systolic blood pressure (**g**), diastolic blood pressure (**h**), and mean arterial blood pressure (**i**) in the high genetic risk score group. PM_10_, particulate matter ≤10 μm. Solid lines, spline curves; shaded area, 95 % confidence interval

**Fig. 2:**
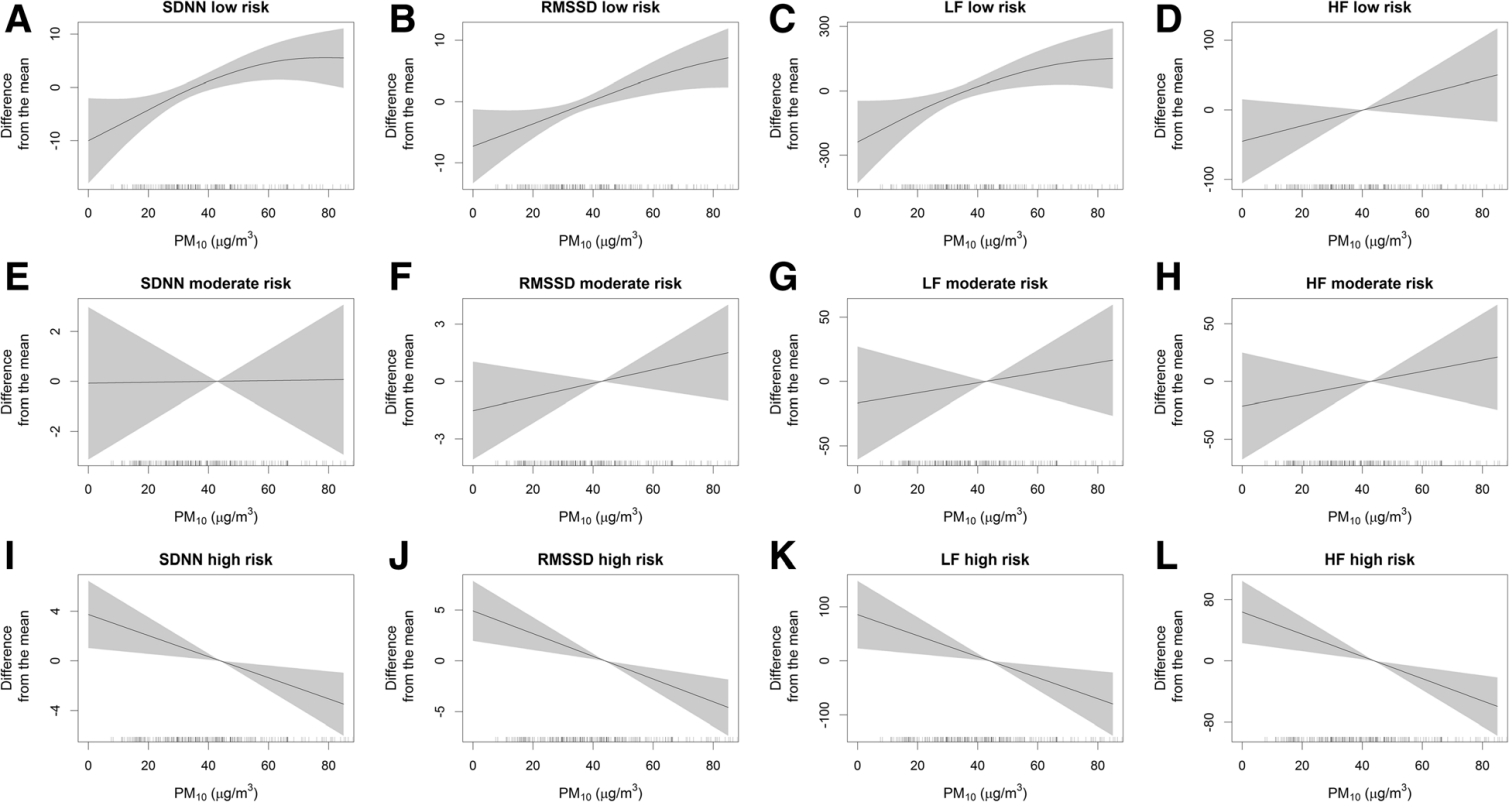
Associations between ambient PM_10_ concentration and heart rate variability in generalized additive mixed models, stratified according to tertiles of genetic risk score for heart rate variability, Korean Elderly Environmental Panel Study, 2008–2010. Models were adjusted for age, sex, smoking status, alcohol drinking, body mass index, hypertension medication, and apparent temperature. The plots show the associations of ambient PM_10_ concentration with SDNN (**a**), RMSSD (**b**), LF (**c**), and HF (**d**) in the low genetic risk score group; the associations with SDNN (**e**), RMSSD (**f**), LF (**g**), and HF (**h**) in the moderate genetic risk score group; and the associations with SDNN (**i**), RMSSD (**j**), LF (**k**), and HF (**l**) in the high genetic risk score group. HF, high frequency power for frequency domain; LF, low frequency power for frequency domain; PM_10_, particulate matter ≤10 μm; RMSSD, root mean square of successive differences for time domain; SDNN, standard deviations of normal-to-normal intervals for time domain. Solid lines, spline curves; shaded area, 95 % confidence interval

We conducted nonparametric analyses using generalized additive mixed models to assess the association of air pollution exposure with blood pressure and heart rate variability within each genetic risk score tertile (low: 6–11 risk alleles, moderate: 12–13, high: 14–19 for blood pressure; low: 8–12, moderate: 13–14, high: 15–21 for heart rate variability). The directions of the associations were different between the low risk group and the high risk group, especially for heart rate variability (Figs. 1 and 2, see Additional file 1: Figures S1–4). The associations of air pollution exposure with blood pressure and heart rate variability were modified by the genetic risk scores for the corresponding outcomes (see Additional file 1: Table S1 and Additional file 1: Table S2). However, the genetic risk scores themselves were not associated with blood pressure or heart rate variability (data not shown).

Several sensitivity analyses revealed robust results. First, we observed very similar results for the interaction testing of each SNP using the 2-df joint test. Second, we did not observe any appreciable change when we selected different sets of SNPs from each linkage disequilibrium pair and constructed alternative genetic risk scores. Third, inverse probability weighting for the follow-up observations caused no appreciable changes (data not shown).

## Discussion

In the present study, air pollution exposure was positively associated with blood pressure among non-institutionalized elderly participants. Although we did not observe an association between air pollution exposure and heart rate variability among the whole study population, air pollution exposure was inversely associated with heart rate variability in the high genetic risk group, after we stratified the participants according to genetic risk score which was calculated based on the SNPs in oxidative stress genes. The associations of air pollution exposure with blood pressure and heart rate variability were modified by the genetic risk scores, suggesting that the associations are mediated, at least partly, by oxidative stress pathways.

A limited number of studies have investigated the interactions between air pollution exposure and genetic polymorphisms with regard to blood pressure or heart rate variability. Moreover, these studies were conducted in a few study populations that consisted of middle-aged or elderly Caucasians. The association between black carbon exposure and blood pressure was reported to be modified by polymorphisms in microRNA processing genes [18], but not by polymorphisms in oxidative stress genes [5, 14]. Polymorphisms in several genes including oxidative stress genes have been demonstrated to modify the associations between particulate matter ≤2.5 μm or traffic-related PM_10_ and heart rate variability [11–13, 15–17]. In the present study, which was conducted among an Asian population which is different from Caucasian populations in the distribution of oxidative stress gene polymorphisms and inflammatory marker levels related to cardiovascular disease risk [33, 34], we investigated the interactions between exposure to air pollutants (PM_10_, NO_2_, and SO_2_) and 46 SNPs in 18 oxidative stress genes on blood pressure and heart rate variability. Our results suggest that there is a common oxidative stress-related mechanism that affects the associations of air pollution exposure with blood pressure and heart rate variability.

Among 46 SNPs in 18 oxidative stress-related genes, 15 SNPs in 11 genes showed interactions with air pollutants with regard to blood pressure, and 17 SNPs in 17 genes showed interactions with regard to heart rate variability. The present results suggest that the observed interactions between air pollutants and genetic predisposition to oxidative stress on blood pressure and heart rate variability may be attributable to cumulative effects of various risk alleles and not to specific SNPs. Because we genotyped SNPs exhibiting ≥5 % minor allele frequency for the Japanese and Chinese populations in the HapMap, our results can be considered to support common disease-common variant hypothesis, which is characterized by the condition of high allele frequencies with low relative risk [35]. Despite the methodological difficulties in considering and summarizing different interaction effects among SNPs in the gene-environment interaction analysis and assessing potential gene-gene interactions, the current study has originality in investigating gene-environment interactions rather than main effects of genetic polymorphisms.

Previous studies have reported heterogeneous results regarding the association between air pollution exposure and heart rate variability, both in terms of its magnitude and its direction [36]. For example, some studies reported that air pollution exposure was associated with a decline in heart rate variability [37, 38], whereas other studies have reported a null association or an association with an increase in heart rate variability [39, 40]. These inconsistencies might be attributable to differences in the study populations’ genetic characteristics, and especially in genes that are related to oxidative stress. In the present study, we observed opposite directions for the associations among participants with high and low genetic risk scores calculated using their oxidative stress gene polymorphisms. These opposing directions might explain why we did not observe an association between air pollution exposure and heart rate variability among the whole study population.

The mechanism for the positive association between air pollution exposure and heart rate variability in the low genetic risk group is not clear. Previous studies have suggested that low-dose oxidative stress that is induced by environmental pollutants could stimulate an beneficial adaptive effect, by increasing the synthesis of antioxidants and promoting mitochondrial function [41–43]. Although the level of exposure to air pollutants is the same, people with reduced genetic predisposition to oxidative stress might actually benefit from low-dose oxidative stress challenges via air pollution. However, data regarding the protective influence of low-level air pollution exposure are limited [44–46]. Moreover, the role of genetic polymorphisms in this process has not been investigated. Further studies are needed to fully understand the gene-environment interactions that we observed.

The associations of the increases in interquartile range for each air pollutant (PM_10_, NO_2_, and SO_2_) with outcomes of interest were comparable with respect to directions and magnitudes (Table 3). When we constructed multiple pollutant models that include all air pollutants and covariates, SO_2_ levels remained to be associated with higher blood pressure (data not shown). However, this result should be interpreted cautiously because precise assessment of independent contributions of each air pollutant is difficult due to high correlation among individual air pollutants (data not shown).

The gene-environment interaction may also explain why only a small portion of heritability can be explained by common risk variants in genome-wide association studies [9, 47]. In the present study, genetic risk scores were not associated with blood pressure or heart rate variability, although interactions with air pollution exposure were observed. Similarly, air pollution exposure was not associated with heart rate variability when we analyzed the whole study population. However, when we stratified the study population according to genetic risk, air pollution exposure was inversely associated with heart rate variability in the high genetic risk group. Our results suggest that genetic or environmental factors cannot be accurately evaluated if only the main effects are considered without considering potential interactions.

The present study has several limitations. First, we used monitoring data as a proxy for individual air pollution exposures, which introduces the possibility of misclassification. However, estimating the individual exposure from the monitoring data based on the participant’s residence appears to be reasonable, given the fact that most of the participants were retired or unemployed due to their age. Second, the present study was conducted among elderly adults and our findings may not generalize to younger populations. Third, although we evaluated a relatively large number of oxidative stress-related genetic polymorphisms, some studies have reported that non-oxidative stress-related genes might also interact with air pollution exposure with regard to blood pressure or heart rate variability [1, 16, 18].

## Conclusions

We found that the associations of air pollution exposure with blood pressure and heart rate variability were modified by genetic risk scores calculated using polymorphisms in oxidative stress genes, which suggest that an oxidative stress-related mechanism may contribute to the associations. Because the effects of air pollution exposure may differ by genetic predispositions, future air quality guidelines should take into account genetically susceptible populations and standards should be set to the lower levels considering the potential adverse health effects in these vulnerable subgroups.

## Additional file

Additional file 1: Table S1. Association of PM_10_, NO_2_, and SO_2_ With Blood Pressure per Interquartile Range Increase in Air Pollutant Concentration, Stratified According to Tertiles of Genetic Risk Score for Blood Pressure, Korean Elderly Environmental Panel Study, 2008–2010. **Table S2.** Association of PM_10_, NO_2_, and SO_2_ with Heart Rate Variability per Interquartile Range Increase in Air Pollutant Concentration, Stratified According to Tertiles of Genetic Risk Score for Heart Rate Variability, Korean Elderly Environmental Panel Study, 2008–2010. **Figure S1.** Associations between ambient NO_2_ concentration and blood pressure in generalized additive mixed models, stratified according to tertiles of genetic risk score for blood pressure, Korean Elderly Environmental Panel Study, 2008–2010. **Figure S2.** Associations between ambient SO_2_ concentration and blood pressure in generalized additive mixed models, stratified according to tertiles of genetic risk score for blood pressure, Korean Elderly Environmental Panel Study, 2008–2010. **Figure S3.** Associations between ambient NO_2_ concentration and heart rate variability in generalized additive mixed models, stratified according to tertiles of genetic risk score for heart rate variability, Korean Elderly Environmental Panel Study, 2008–2010. **Figure S4.** Associations between ambient SO_2_ concentration and heart rate variability in generalized additive mixed models, stratified according to tertiles of genetic risk score for heart rate variability, Korean Elderly Environmental Panel Study, 2008–2010. (DOCX 634 kb)

## Abbreviations

BP: blood pressure
HF: high frequency power for frequency domain
HRV: heart rate variability
LF: low frequency power for frequency domain
PM_10_: particulate matter ≤10 μm
RMSSD: root mean square of successive differences for time domain
SDNN: standard deviations of normal-to-normal intervals for time domain
SNP: single-nucleotide polymorphism
