# Supplementary material for: Associations of air pollution exposure with blood pressure and heart rate variability are modified by oxidative stress genes: A repeated-measures panel among elderly urban residents
Source: Environ Health. 2016 Mar 25;15:47. doi: 10.1186/s12940-016-0130-3 (PMC4807581; doi:10.1186/s12940-016-0130-3)
Supplement: Additional file 1: Table S1. — Association of PM10, NO2, and SO2 With Blood Pressure per Interquartile Range Increase in Air Pollutant Concentration, Stratified According to Tertiles of Genetic Risk Score for Blood Pressure, Korean Elderly Environmental Panel Study, 2008–2010. Table S2. Association of PM10, NO2, and SO2 with Heart Rate Variability per Interquartile Range Increase in Air Pollutant Concentration, Stratified According to Tertiles of Genetic Risk Score for Heart Rate Variability, Korean Elderly Environmental Panel Study, 2008–2010. Figure S1. Associations between ambient NO2 concentration and blood pressure in generalized additive mixed models, stratified according to tertiles of genetic risk score for blood pressure, Korean Elderly Environmental Panel Study, 2008–2010. Figure S2. Associations between ambient SO2 concentration and blood pressure in generalized additive mixed models, stratified according to tertiles of genetic risk score for blood pressure, Korean Elderly Environmental Panel Study, 2008–2010. Figure S3. Associations between ambient NO2 concentration and heart rate variability in generalized additive mixed models, stratified according to tertiles of genetic risk score for heart rate variability, Korean Elderly Environmental Panel Study, 2008–2010. Figure S4. Associations between ambient SO2 concentration and heart rate variability in generalized additive mixed models, stratified according to tertiles of genetic risk score for heart rate variability, Korean Elderly Environmental Panel Study, 2008–2010. (DOCX 634 kb) [file 12940_2016_130_MOESM1_ESM.docx]

**Methods**

*Statistical model> linear mixed models for assessing interactions between air pollutants and SNPs*

The general form of the model was

Y*_ij_* = β_0_ + β_1_Air pollutant*_ij_* + β_2_SNP*_i_* + β_3_(Air pollutant*_ij_* × SNP*_i_*) + β_4_Z*_ij_* + b*_0i_* + e*_ij_*,

where, for the *i*th individual at the *j*th observation, Y*_ij_* is blood pressure or log-transformed heart rate variability, Air pollutant_ij_ is observed air pollutant level on daily lag, Air pollutant*_ij_* × SNP*_i_* is the cross-product term between air pollutant and SNP for specific individual, Z*_ij_* is a vector of covariates, and b*_0i_* is a random intercept.

*Statistical model> generalized additive mixed models*

The general form of the generalized additive mixed model was

Y*_ij_* = β_0_ + β_1_S(Air pollutant*_ij_*) + β_2_SNP*_i_* + β_3_Z*_ij_* + b*_0i_* + e*_ij_*,

where, for the *i*th individual at the *j*th observation, Y*_ij_* is blood pressure or log-transformed heart rate variability, S(.) is a smooth function, Air pollutant*_ij_* is an air pollutant level on daily lag, SNP*_i_* is the SNP of specific individual, Z*_ij_* is a vector of covariates, and b*_0i_* is a random intercept.

**Table S1.** Association of PM_10_, NO_2_, and SO_2_ With Blood Pressure per Interquartile Range Increase in Air Pollutant Concentration, Stratified According to Tertiles of Genetic Risk Score for Blood Pressure, Korean Elderly Environmental Panel Study, 2008–2010

|  |  | PM_10_ |  |  | NO_2_ |  |  | SO_2_ |  |  |
| --- | --- | --- | --- | --- | --- | --- | --- | --- | --- | --- |
| Genetic risk score |  | estimate | 95% CI | *P*-int | estimate | 95% CI | *P*-int | estimate | 95% CI | *P*-int |
| Low | SBP | -0.02 | -1.44, 1.41 | 0.0453 | -0.19 | -1.67, 1.28 | 0.0224 | 1.28 | -0.34, 2.91 | 0.0038 |
|  | DBP | -0.06 | -0.91, 0.79 | 0.0564 | 0.03 | -0.85, 0.91 | 0.0810 | 0.40 | -0.55, 1.35 | 0.0804 |
|  | MAP | -0.02 | -1.01, 0.97 | 0.0385 | -0.04 | -1.07, 0.98 | 0.0353 | 0.68 | -0.44, 1.80 | 0.0170 |
| Moderate | SBP | 0.90 | -0.14, 1.95 |  | 0.88 | -0.36, 2.13 |  | 0.94 | -0.28, 2.16 |  |
|  | DBP | 0.75 | 0.10, 1.41 |  | 0.87 | 0.11, 1.63 |  | 0.61 | -0.15, 1.38 |  |
|  | MAP | 0.82 | 0.08, 1.56 |  | 0.89 | 0.02, 1.76 |  | 0.69 | -0.17, 1.55 |  |
| High | SBP | 2.33 | 0.75, 3.91 |  | 2.85 | 1.03, 4.66 |  | 3.80 | 1.88, 5.72 |  |
|  | DBP | 1.70 | 0.79, 2.62 |  | 1.94 | 0.88, 2.99 |  | 1.66 | 0.51, 2.81 |  |
|  | MAP | 1.87 | 0.80, 2.95 |  | 2.20 | 0.97, 3.44 |  | 2.33 | 1.00, 3.67 |  |

DBP, diastolic blood pressure; MAP, mean arterial pressure; NO_2_, nitrogen dioxide; *P*-int, *P* value for interaction; PM_10_, particulate matter ≤10 µm; SBP, systolic blood pressure; SO_2_, sulfur dioxide.

The linear mixed models were adjusted for age, sex, smoking status, alcohol drinking, body mass index, hypertension medication, and apparent temperature. Associations with blood pressure are shown for PM_10_ and NO_2_ on lag day 1 and SO_2_ on lag day 3.

**Table S2.** Association of PM_10_, NO_2_, and SO_2_ with Heart Rate Variability per Interquartile Range Increase in Air Pollutant Concentration, Stratified According to Tertiles of Genetic Risk Score for Heart Rate Variability, Korean Elderly Environmental Panel Study, 2008–2010

|  |  | PM_10_ |  |  | NO_2_ |  |  | SO_2_ |  |  |
| --- | --- | --- | --- | --- | --- | --- | --- | --- | --- | --- |
| Genetic risk score |  | estimate | 95% CI | *P*-int | estimate | 95% CI | *P*-int | estimate | 95% CI | *P*-int |
| Low | SDNN | 0.13 | 0.07, 0.19 | <0.0001 | 0.10 | 0.03, 0.16 | 0.0004 | 0.04 | -0.02, 0.11 | 0.0066 |
|  | RMSSD | 0.19 | 0.12, 0.27 | <0.0001 | 0.10 | 0.01, 0.18 | 0.0003 | 0.03 | -0.05, 0.11 | 0.0120 |
|  | LF | 0.31 | 0.16, 0.46 | <0.0001 | 0.22 | 0.06, 0.39 | 0.0012 | 0.04 | -0.11, 0.19 | 0.0611 |
|  | HF | 0.21 | 0.06, 0.35 | 0.0005 | 0.13 | -0.02, 0.29 | 0.0052 | -0.05 | -0.19, 0.09 | 0.0409 |
| Moderate | SDNN | 0.02 | -0.03, 0.06 |  | 0.02 | -0.03, 0.08 |  | 0.06 | 0.01, 0.11 |  |
|  | RMSSD | 0.02 | -0.03, 0.08 |  | 0.06 | -0.01, 0.12 |  | 0.08 | 0.02, 0.14 |  |
|  | LF | 0.04 | -0.07, 0.16 |  | 0.05 | -0.09, 0.19 |  | 0.16 | 0.05, 0.28 |  |
|  | HF | 0.06 | -0.05, 0.18 |  | 0.09 | -0.06, 0.24 |  | 0.15 | 0.02, 0.27 |  |
| High | SDNN | -0.05 | -0.09, -0.01 |  | -0.08 | -0.12, -0.03 |  | -0.05 | -0.10, -0.01 |  |
|  | RMSSD | -0.07 | -0.12, -0.02 |  | -0.10 | -0.16, -0.04 |  | -0.05 | -0.11, 0.004 |  |
|  | LF | -0.09 | -0.20, 0.02 |  | -0.18 | -0.31, -0.04 |  | -0.07 | -0.20, 0.05 |  |
|  | HF | -0.11 | -0.21, -0.01 |  | -0.17 | -0.29, -0.04 |  | -0.14 | -0.26, -0.02 |  |

HF, high frequency power for frequency domain; LF, low frequency power for frequency domain; NO_2_, nitrogen dioxide; *P*-int, *P* value for interaction; PM_10_, particulate matter ≤10 µm; RMSSD, root mean square of successive differences for time domain; SDNN, standard deviations of normal-to-normal intervals for time domain; SO_2_, sulfur dioxide.

The linear mixed models were adjusted for age, sex, smoking status, alcohol drinking, body mass index, hypertension medication, and apparent temperature. Associations with heart rate variability are shown for PM_10_ on lag day 0, NO_2_ on lag day 1, and SO2 on lag day 2.

**Figure Legends**

**Figure S1.** Associations between ambient NO_2_ concentration and blood pressure in generalized additive mixed models, stratified according to tertiles of genetic risk score for blood pressure, Korean Elderly Environmental Panel Study, 2008–2010.


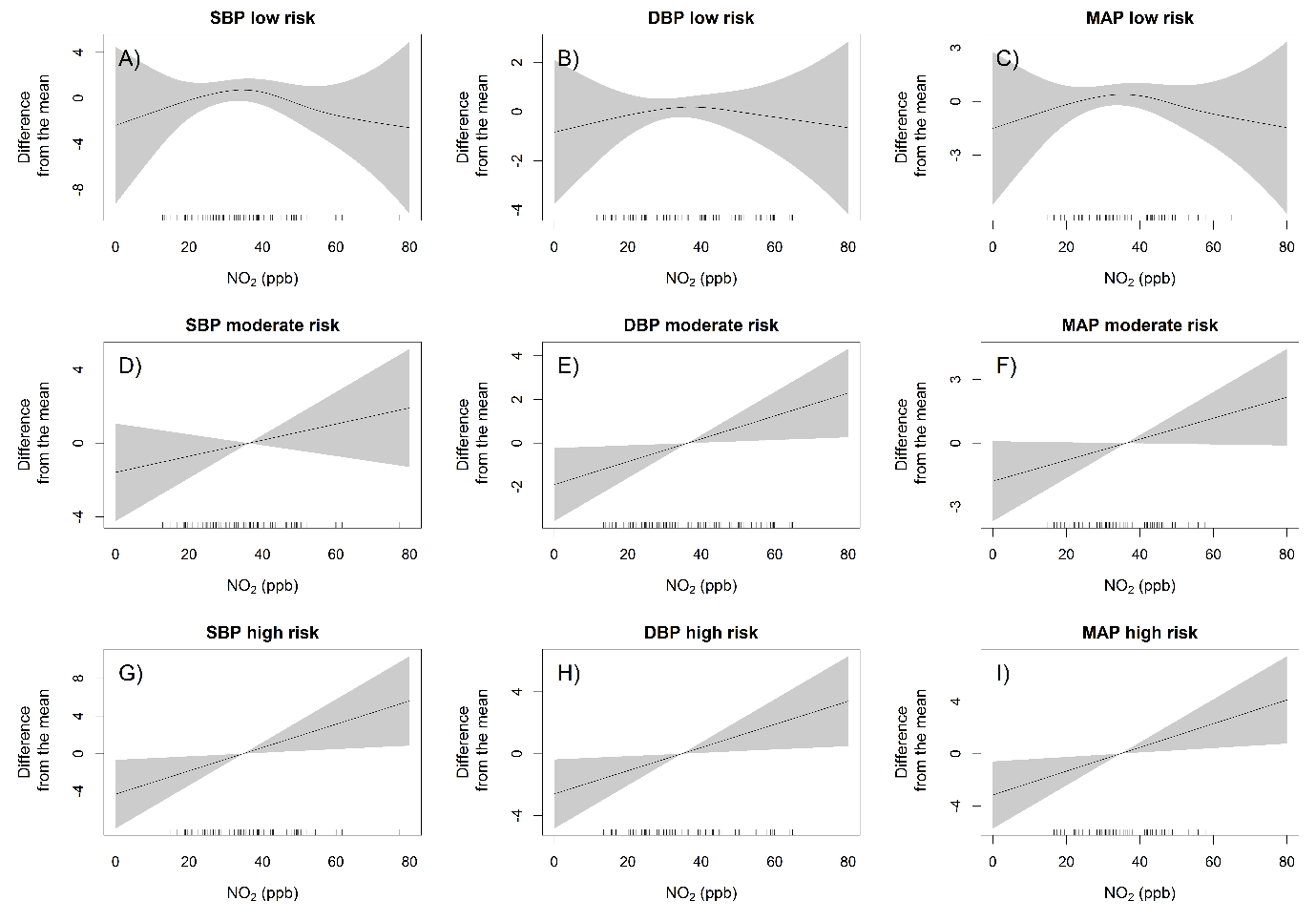


Models were adjusted for age, sex, smoking status, alcohol drinking, body mass index, hypertension medication, and apparent temperature. The plots show the associations of ambient NO_2_ concentration with systolic blood pressure (A), diastolic blood pressure (B), and mean arterial blood pressure (C) in the low genetic risk score group; the associations with systolic blood pressure (D), diastolic blood pressure (E), and mean arterial blood pressure (F) in the moderate genetic risk score group; and the associations with systolic blood pressure (G), diastolic blood pressure (H), and mean arterial blood pressure (I) in the high genetic risk score group. NO_2_, nitrogen dioxide. Solid lines, spline curves; shaded area, 95% confidence interval.

**Figure S2.** Associations between ambient SO_2_ concentration and blood pressure in generalized additive mixed models, stratified according to tertiles of genetic risk score for blood pressure, Korean Elderly Environmental Panel Study, 2008–2010.


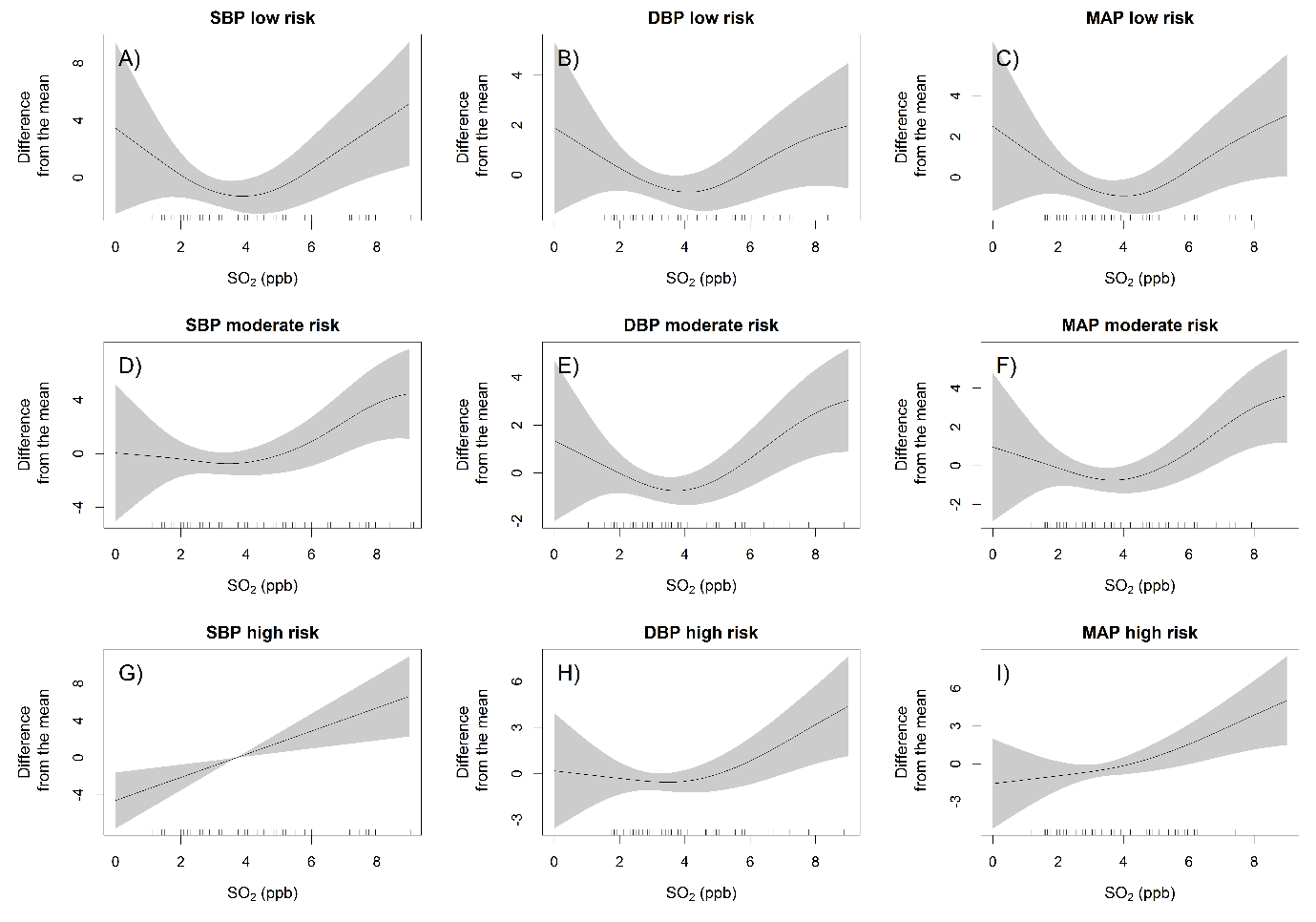


Models were adjusted for age, sex, smoking status, alcohol drinking, body mass index, hypertension medication, and apparent temperature. The plots show the associations of ambient SO_2_ concentration with systolic blood pressure (A), diastolic blood pressure (B), and mean arterial blood pressure (C) in the low genetic risk score group; the associations with systolic blood pressure (D), diastolic blood pressure (E), and mean arterial blood pressure (F) in the moderate genetic risk score group; and the associations with systolic blood pressure (G), diastolic blood pressure (H), and mean arterial blood pressure (I) in the high genetic risk score group. SO_2_, sulfur dioxide. Solid lines, spline curves; shaded area, 95% confidence interval.

**Figure S3.** Associations between ambient NO_2_ concentration and heart rate variability in generalized additive mixed models, stratified according to tertiles of genetic risk score for heart rate variability, Korean Elderly Environmental Panel Study, 2008–2010.


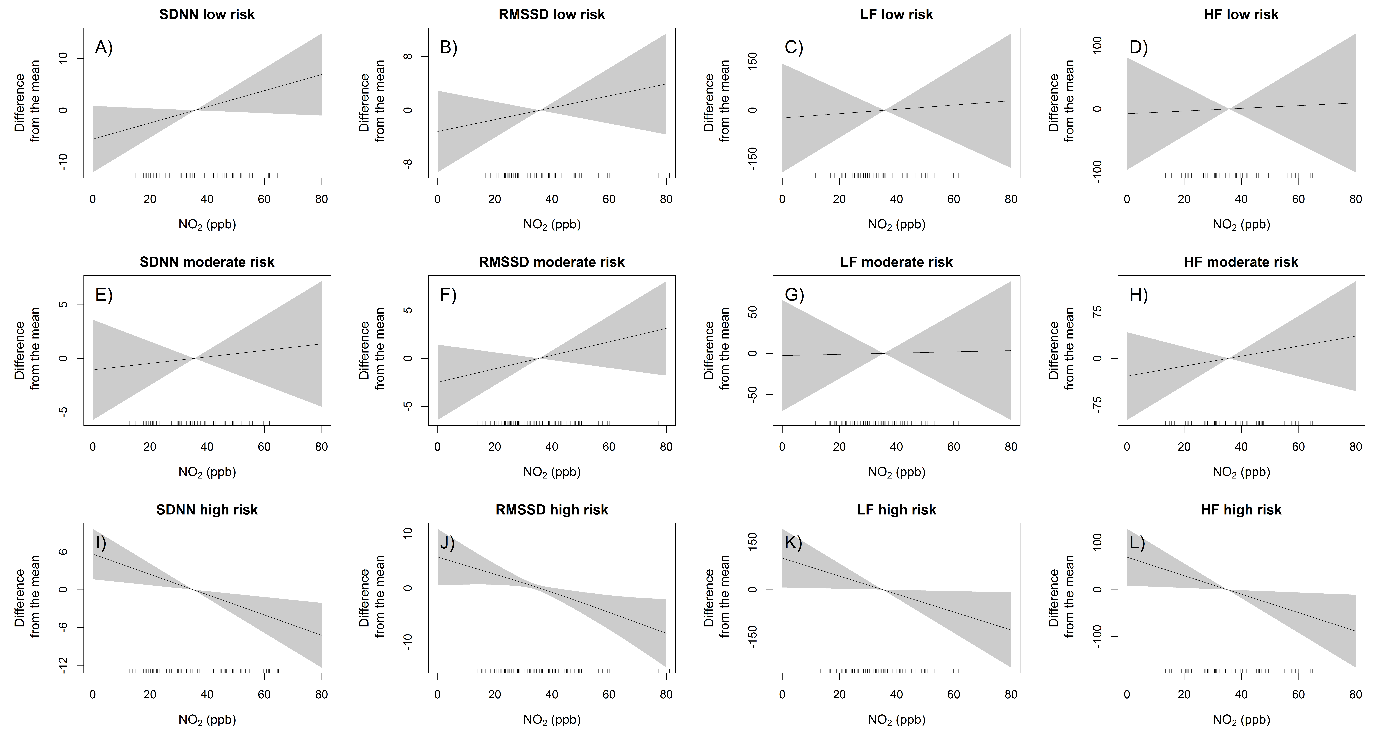


Models were adjusted for age, sex, smoking status, alcohol drinking, body mass index, hypertension medication, and apparent temperature. The plots show the associations of ambient NO_2_ concentration with SDNN (A), RMSSD (B), LF (C), and HF (D) in the low genetic risk score group; the associations with SDNN (E), RMSSD (F), LF (G), and HF (H) in the moderate genetic risk score group; and the associations with SDNN (I), RMSSD (J), LF (K), and HF (L) in the high genetic risk score group. HF, high frequency power for frequency domain; LF, low frequency power for frequency domain; NO_2_, nitrogen dioxide; RMSSD, root mean square of successive differences for time domain; SDNN, standard deviations of normal-to-normal intervals for time domain. Solid lines, spline curves; shaded area, 95% confidence interval.

**Figure S4.** Associations between ambient SO_2_ concentration and heart rate variability in generalized additive mixed models, stratified according to tertiles of genetic risk score for heart rate variability, Korean Elderly Environmental Panel Study, 2008–2010.


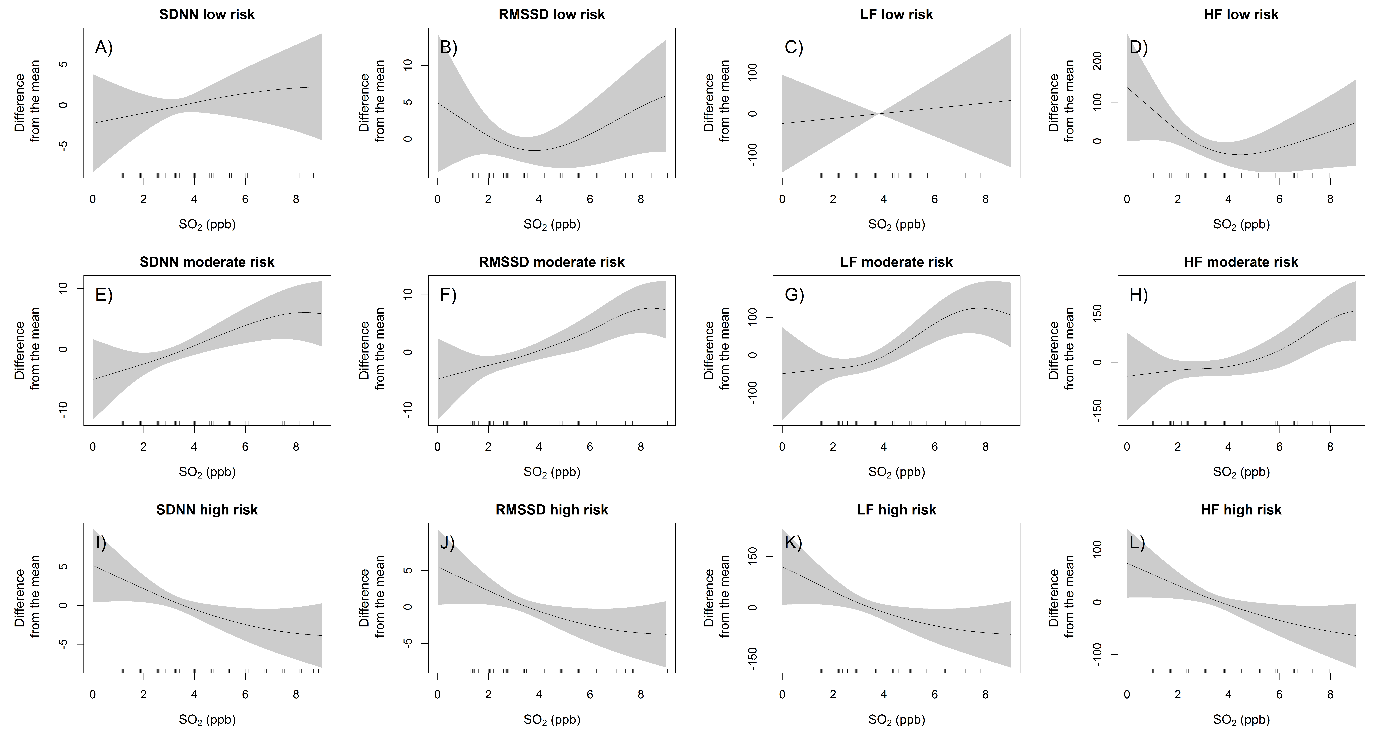


Models were adjusted for age, sex, smoking status, alcohol drinking, body mass index, hypertension medication, and apparent temperature. The plots show the associations of ambient SO_2_ concentration with SDNN (A), RMSSD (B), LF (C), and HF (D) in the low genetic risk score group; the associations with SDNN (E), RMSSD (F), LF (G), and HF (H) in the moderate genetic risk score group; and the associations with SDNN (I), RMSSD (J), LF (K), and HF (L) in the high genetic risk score group. HF, high frequency power for frequency domain; LF, low frequency power for frequency domain; SO_2_, sulfur dioxide; RMSSD, root mean square of successive differences for time domain; SDNN, standard deviations of normal-to-normal intervals for time domain. Solid lines, spline curves; shaded area, 95% confidence interval.
